# Biophysical Reviews contribution call: an issue focus on the life and works of Prof. Har Gobind Khorana on the occasion of the 100th anniversary of the year of his birth

**DOI:** 10.1007/s12551-022-00958-2

**Published:** 2022-05-23

**Authors:** Gopala Krishna Aradhyam, Naranamangalam R. Jagannathan

**Affiliations:** 1 Department of Biotechnology, Bhupat and Jyoti Mehta School of Biosciences, Indian Institute of Technology Madras, Chennai, 600036 India; 2 Department of Radiology, Chettinad Hospital and Research Institute, Kelambakkam, TN 603103 India; 3 Department of Electrical Engineering, Indian Institute of Technology Madras, Chennai, 600036 India

Har Gobind Khorana (1922–2011) received the 1968 Nobel Prize for Physiology and Medicine (together with Marshall W. Nirenberg and Robert W. Holley) for his work in deducing the codon basis of the genetic code (Khorana et al. 1966; Khorana 1968). He later went on to make major contributions to research in membrane protein biochemistry (Khorana 1993).

Starting from humble beginnings as the son of a tax clerk in Raipur, Punjab, Har Gobind Khorana was drawn to science at an early age. With chemistry as his background, he chased and won the biggest race in biology. Gobind (as he was fondly called) carried a smile across the world, even in tough times. Ever endearing, he performed extraordinary research in chemistry and biology, such that the world still benefits greatly from his scientific achievements to this day. To those he interacted with, Gobind set an example on how one can be both an excellent scientist and a compassionate human being—he was indeed open to sharing everything he knew and generated.

This commentary is a call for submissions to celebrate the genius of Gobind on the occasion of the 100th anniversary of his birth. Submitted works are to be published in an upcoming Biophysical Reviews’ Issue Focus on the science and life of Prof. Har Gobind Khorana. Though many articles have already been solicited through personal invitations, international scientists sharing a common area of research interest with Gobind are also encouraged to contact the Guest Editors to discuss the possibility of making a submission. The issue focus will be prepared and edited by the current authors (G.K. Aradhyam, N.R. Jagannathan).

## Submission deadline

Review articles are requested to be submitted prior to August 1st of 2022 with online publication scheduled for Issue 5 of that same year. When submitting your article, please choose the correct Special Issue assignment.

Journal homepage http://www.springer.com/journal/12551.

Online submission https://www.editorialmanager.com/brev/ journal social media.

Biophysical Reviews publishes two types of review formats. A short review (3000–4000 words, 3 figures) or a long review (10,000 words, 10 figures). A series of Commentaries (200–400 words, 1 figure, ~10 references) from scientists having a close relationship with Prof. Har Gobind Khoran are also requested.

## Social media

The journal operates two social media sites, the details of which can be found here:

Twitter™: @BiophysicalRev1

YouTube™: https://www.youtube.com/channel/UCzG_5MWmnrB2UBibtxs2DuA

## Publication cost

Biophysical Reviews is a hybrid open-access journal and therefore operates on a two-track payment. The first track is completely free but requires that the article exists behind a paywall for 6 to 12 months whilst the second track involves an open access charge of ~2000 Euros. Both tracks give a highly desirable publishing outcome for the author. Please contact the Special Issue Editors for further description of this point.
